# Understanding Action and Adventure Sports Participation—An Ecological Dynamics Perspective

**DOI:** 10.1186/s40798-017-0084-1

**Published:** 2017-04-26

**Authors:** Tuomas Immonen, Eric Brymer, Dominic Orth, Keith Davids, Francesco Feletti, Jarmo Liukkonen, Timo Jaakkola

**Affiliations:** 1 Faculty of Sport and Health Sciences, University of Jyväskylä, Jyväskylä, Finland; 2 Institute of Sport, Physical Activity and Leisure, Leeds Beckett University, Leeds, UK; 3 Faculty of Behavioral and Movement Sciences, MOVE Research Institute, Vrije Universiteit, Amsterdam, The Netherlands; 4 Centre for Sports Engineering Research, Sheffield Hallam University, Sheffield, UK; 5 S.Maria delle Croci Hospital Ravenna, Ravenna, Italy; 6 Department of Electronics Information and Bioengineering, Politecnico di Milano University Milan, Milan, Italy; 7 Faculty of Sport and Health Sciences, University of Jyväskylä, Jyväskylä, Finland

## Abstract

Previous research has considered action and adventure sports using a variety of associated terms and definitions which has led to confusing discourse and contradictory research findings. Traditional narratives have typically considered participation exclusively as the pastime of young people with abnormal characteristics or personalities having unhealthy and pathological tendencies to take risks because of the need for thrill, excitement or an adrenaline ‘rush’. Conversely, recent research has linked even the most extreme forms of action and adventure sports to positive physical and psychological health and well-being outcomes. Here, we argue that traditional frameworks have led to definitions, which, as currently used by researchers, ignore key elements constituting the essential merit of these sports. In this paper, we suggest that this lack of conceptual clarity in understanding cognitions, perception and action in action and adventure sports requires a comprehensive explanatory framework, ecological dynamics which considers person-environment interactions from a multidisciplinary perspective. Action and adventure sports can be fundamentally conceptualized as activities which flourish through creative exploration of novel movement experiences, continuously expanding and evolving beyond predetermined environmental, physical, psychological or sociocultural boundaries. The outcome is the emergence of a rich variety of participation styles and philosophical differences within and across activities. The purpose of this paper is twofold: (a) to point out some limitations of existing research on action and adventure sports; (b) based on key ideas from emerging research and an ecological dynamics approach, to propose a holistic multidisciplinary model for defining and understanding action and adventure sports that may better guide future research and practical implications.

## Key Points

- Current frameworks and definitions in the study of action and adventure sports have led to unclear discourse and contradictory research findings.
- Ecological dynamics provides a holistic, multidisciplinary platform for comprehensive and nuanced understanding of action and adventure sports participation.
- More research should be directed towards understanding the relationship of sociocultural affordances and creative and innovative actions of individuals and groups, expressed with various emerging participation styles and philosophies.

## Background

Participant rates in *action and adventure sports* (AAS) are on the rise, surpassing many traditional sports in popularity [1, 2]. In this opinion piece, the term AAS refers to a variety of sports such as snowboarding, surfing, parkour, climbing and BASE jumping.

It could be argued that past research has tended to overemphasize a risk-taking perspective when discussing participation in AAS. This has imposed a superficial lens on participation, viewed as fundamentally influenced by processes underpinned by risk-taking, promoting a biased perspective where AAS are perceived as a context for taking socially unacceptable, pathological and unnecessary risks [3–10]. To exemplify, classic theoretical approaches proposed to explain behavior in AAS include sensation seeking [11, 12], edgework [13, 14], type ‘T’ personality [4] and psychoanalysis [15]. The main narrative of these approaches is that personality traits and socialization processes predispose individuals to participate in risky or life-threatening activities [16].

The overemphasized focus on risk has meant that other important aspects of participation have been widely overlooked [17–19]. The most significant limitation with ‘risk-centric’ approaches is that fundamentally, the activity may be defined as pathological and unhealthy. Most theoretical explanations assume that participation reflects a desire for thrills, excitement or adrenaline-seeking. However, emerging research indicates that these explanations are an oversimplification [18, 20, 21] and do not reflect the lived experience of participants who refute the thrills and adrenaline notion and instead describe AAS participation as meaningful and life-enhancing [16, 18, 19]. In recent years, a growing body of literature supports the idea that participation can promote psychological and physical well-being and health [18, 21–24] in a variety of ways. Research has recently linked even the most extreme forms of AAS to varied physical and psychological health and well-being outcomes. In summary, activities generally provide benefits, such as (1) opportunities to fulfill basic psychological needs of autonomy, competence and relatedness [16], (2) opportunities to overcome challenge [25], (3) opportunities to experience intense emotions [26], (4) increased positive psychological outcomes such as resilience, self-efficacy and positive affect [21, 22, 27], (5) increased physical activity levels [16, 28] and (6) feelings of connection to nature [17, 29, 30]. These benefits are often overlooked as modern societies are increasingly focused on providing ‘safe’ behavioural environments which reduce physical risks for members [16, 31].

One possible explanation for the discrepancies in popular imagination and theoretical explanations in research on AAS participation is that conceptual definitions for these activities remain unclear. Common terms include action sports [32, 33], adventure sports [25, 34, 35], extreme sports [36, 37], lifestyle sports [38–40], alternative sports [41, 42] and (high) risk sports [43–46]. This is a key concern because the lack of widely shared definitions or classifications among researchers has led to confusing discourse and contradictory research findings.

Here, we summarize the lack of clarity of the past research into five main issues: (1) activities requiring high level of self-knowledge, personal skills, training, commitment, environmental knowledge and task knowledge, such as BASE jumping or big-mountain skiing, are assumed to be in the same category as activities that require no previous experience or knowledge of the activity or environment, such as commercial white-water rafting or bungee jumping [39]. Findings from studies on motivations, for instance, in activities such as commercial rafting, may not generalize to understanding participation in activities such as BASE jumping. (2) Research has identified various motives for participation, such as connection with nature [17, 47], relieving boredom [25], pushing personal boundaries and overcoming fear [18, 20], social relationships [25], pleasurable kinesthetic bodily sensations [47], control, mastery and skill [20] and goal achievement [19]. These findings indicate that the spectrum of motives is wide and dependent on the form of activity. (3) Sports differ in terms of activity duration and intensity leading to different interaction effects on behaviour. For example, a mountaineering trip might take weeks, exposing the individual to prolonged periods of environmental uncertainty, whereas other sports, such as BASE jumping, might only take seconds. (4) Another finding, questioning the idea that AAS are synonymous with youth sports or sub-cultures, is that participants represent a broad demographic, including males and females of various age ranges and education and income levels [48]. (5) Also, each sport has its own unique history and development patterns [39]. Many are still evolving and fragmenting. For example, snowboarding can take place in urban environments, formerly the home of skateboarding. At the same time, boundaries between sports are becoming more dynamic, for example with snowboarding also being undertaken at high altitude where conventional snowboarding skills are combined with mountaineering techniques. One potential pitfall in categorizing a specific sport, or a group of sports as a single entity (i.e. as ‘extreme’), could be that distinguishing characteristics of different participation styles and the role of the interactions between individual characteristics of participants and varying performance environments are overlooked.

The term ‘sport’ is often viewed as synonymous with structured competition. However, from an etymological perspective, the English word ‘sport’, derived from old French word ‘desport’, originally refers to a ‘pastime’ [49]. Also, for example the Finnish equivalent ‘urheilu’ is derived from ‘urhea’, referring to the adjectives ‘brave’ or ‘valiant’ and can be defined as an activity to maintain physical fitness, recreation or as a competition according to specific rules [50]. Here, we adopt the broad definition including the dimensions of self-development and recreation. Specifically, sports are considered to be multi-faceted, boundary-crossing activities [51], which do not necessarily involve structured competitive activity, regulated performance environments, rules or institutions.

Understanding these nuances in definition is crucial for recognizing how a variety of sociocultural values have constrained emerging participation styles. Consequently, it is imperative that we develop a clear understanding of what exactly constitutes an AAS. The confusion in current discourse indicates an evident need for a more nuanced, holistic and multidisciplinary approach to the study of AAS [34]. Thus, AAS should be understood through emphasizing a different level of analysis to traditional perspectives.

In this paper, we argue that a more comprehensive understanding of AAS participation can be achieved through the ecological dynamics (ED) framework. This framework promotes an understanding, over relevant timescales (such as performance and learning), of how irregular and unpredictable constraints in AAS influence the emergent dynamics of continuous, dynamical relationships evolving between an individual and her/his environment [34, 52]. The cornerstones of ED are that (a) movement behaviors are examined and understood at the performer-environment scale of analysis; (b) perception of information provides opportunities for action (i.e., affordances) and is the basis of how behaviours are regulated at an individual level; and (3) performance behaviours are self-organized over time under interacting constraints [34, 52, 53]. We discuss how these performance characteristics compose a wide variety of participation styles, shaped by key constraints which effectively support the innovative actions of participants and are linked to individuals’ cognitions, actions and perceptions of diverse physical and sociocultural performance environments [34, 52].

## Discussion

### How the Ecological Dynamics Framework can Address Emergent Characteristics of Participation in AAS

At a fundamental level, a common feature in AAS is that they are highly dependent on demands that emerge as the participant engages with the environment, when factors external to a performer play a major role on emergent behaviours [17, 29, 32]. In addition, there is a tendency for AAS to be continuously adapted across different activity boundaries. An advantage of adopting an emergent perspective (ED) in defining sports is that it easily captures the constant evolution of new techniques, variations in participation styles and philosophies and the continuous striving for dynamic creativity and innovation. For example, institutionalization and professionalization of AAS has led to a shift in emphasis in how these sports are occasionally perceived, transitioning from ‘youth sub-culture’ to ‘competitive achievement’ sports now included in traditional organized events such as the Olympic Games [30, 53]. Also, activities characterized as self-directed and conducted in nature are increasingly being repackaged as more structured, formalized, indoor leisure experiences [30]. The desired, independent experience and relatively unpredictable outcome is paradoxically being sold as a predictable, managed commodity [54]. To clarify, we propose AAS to be clearly understood as sports which do not necessarily involve a strictly defined competitive structure, regulated by institutions. They are, however, differentiated from commodities in that participation requires specific skills, which are dynamic in form and continuously subject to constraints of innovation, refinement and creativity by participants. Thus, their very essence is predicated on continuous evolution and development in previously unseen ways and environments, when ‘established’ movement patterns are constantly complemented with emerging, novel techniques and creative styles of action [34, 52]. This striving clearly requires the continuous and intertwined relationship between cognition, perception and action [55, 56].

#### Constraints on AAS Participation

**Fig. 1:**
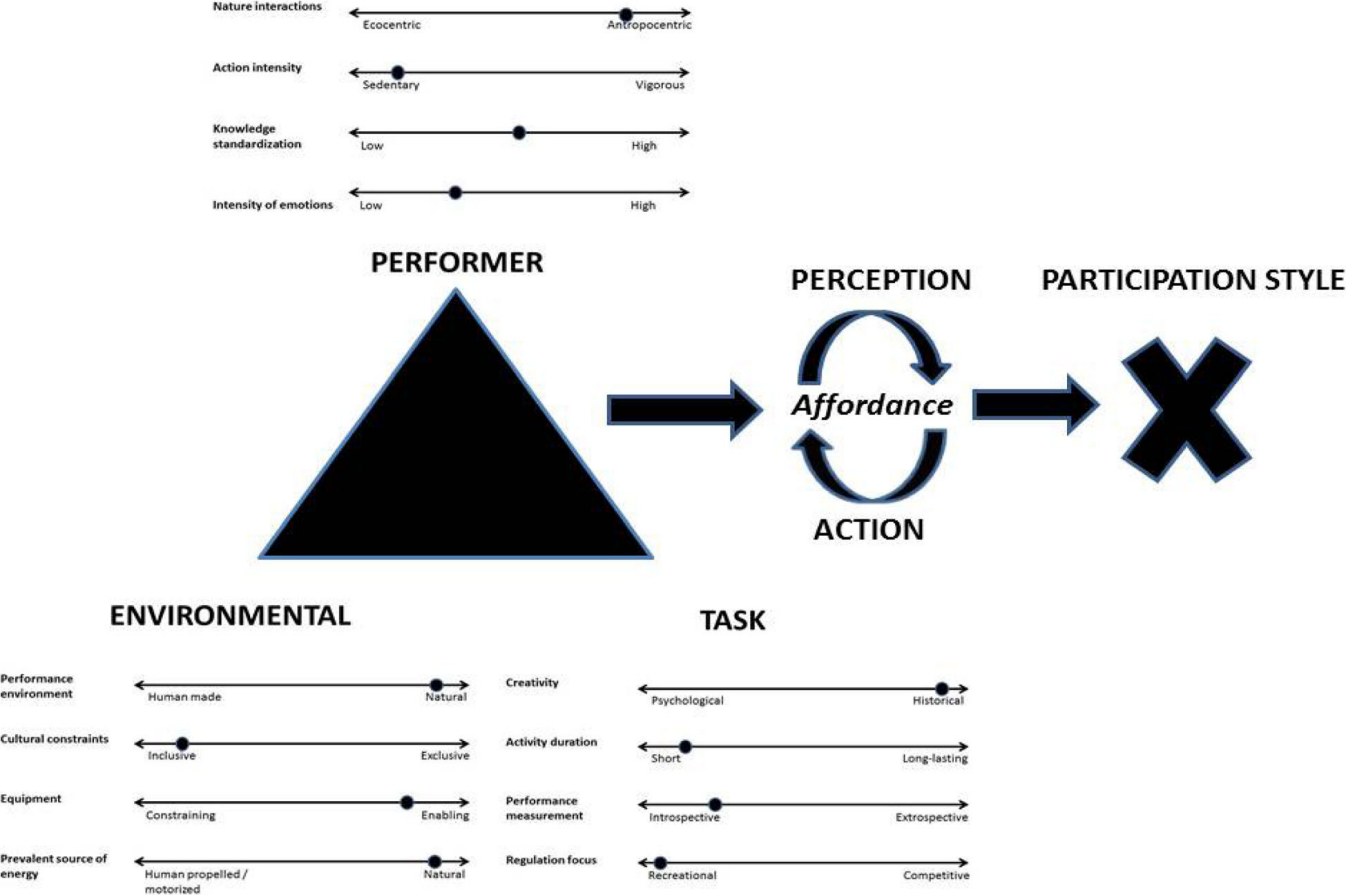
Interacting constraints on action and adventure sports participation. Figure 1 was derived from ideas of Newell [54] and Davids, Button and Bennett [53]. Emerging, adaptive performer-environment relationships are scaled by situational and context-dependent key constraints that, in combination, define an AAS form of life/participation style. Due to the infinite combinations and complexity of interactions, irregular and unpredictable constraints are illustrated on continua, attending to significant situational and contextual fluctuations in each participant’s experience. Included in the figure are exemplary constraints, as reported in existing literature [16, 18, 21, 22, 25, 26, 29, 30, 34, 36, 45, 47, 63, 72–78], and varying characteristics and performance environments of AAS in their current states of development. For better understanding of AAS participation, new emerging constraints should be a posteriori included in the model according to complementary research findings and constant evolution of AAS

Key constraints [54] (Fig. 1) that shape individuals’ cognitions, actions and decision-making processes have been well examined in traditional sports such as soccer, basketball and cricket [55–57].

*Individual constraints* include structural (e.g. height, weight, body shape, technical abilities, connectivity of synapses in the brain) and psychological (e.g. motivations, emotions, cognitions) characteristics of an athlete [34, 53, 57]. Recent studies on AAS have indicated that psychological factors such as fear, anxiety, beliefs and motivation have significant roles during participation and considerable effect on how the environment is perceived by individuals [21, 58, 59]. For example, experiencing varying intensities of fear can trigger panic and impair performance, but it can also be seen as a crucial component of effective performance and positive and potentially life-changing experiences [60].

*Task constraints* in traditional sports include specific rules, task goals and instructional constraints [34]. AAS are not primarily controlled in the same way as mainstream sports by organizational frameworks, regulated competitive structures or strictly governed rules [34, 61]. They, therefore, differ from many sports in that they are not usually characterized by the traditional concept of rule-based task constraints.

*Environmental constraints* are physical (e.g. weather, gravity, ambient light) or sociocultural (e.g. values, family support, cultural norms) [34]. Juxtaposed to purely competitive sports, AAS offer a possibility for mastery and perfection in relation to challenging environments [25, 34, 35]. Unpredictable constraints vary in relation to performances taking place on land, on air, on water, either afloat or submerged, and can be natural (e.g. alpine terrains), partially artificial (e.g. shoveled ‘backcountry kickers’ in snowboarding) or entirely artificial (e.g. skateboard parks). In addition to gravity-driven (e.g. BASE jumping) and human-propelled (e.g. parkour, climbing) forms of movement, the dominant source of energy for action can be exploited in natural phenomena such as an ocean swell in surfing or a thermal updraft in BASE jumping (Fig. 1).

#### AAS as a ‘Form of Life’

*Affordances* refer to aspects of the environment perceived on the basis of what they offer, invite or demand in terms of action [62]. Different surfaces, substances, objects or other individuals in the environment can afford different actions from different people in relation to individual constraints [53, 62]. *Effectivities* are complementary capabilities of an individual that can realize affordances in coherent forms of behaviour [63, 64]. For example, skateboarders might seek handrails in urban environments as an affordance to creatively perform, normally perceived to support locomotion by other individuals, and a skilled skateboarder might approach the same handrail with different set of ‘tricks’ compared to novice.

Wittgenstein’s [65, 66] concept of the ‘form of life’ implies how sociocultural practices of humans can constrain the emergence of specific behavioral patterns [63, 67], emphasizing that effectivities are not merely relative to particular individuals actually perceiving or detecting affordances but also have an existence relative to the skills available in practice, or to the abilities available in a form of life as a whole [67]. For example, the same beach and wave conditions can afford many surfing styles: ‘classic’ walking maneuvers on a longboard or more radical direction changes on a high-performance shortboard. Therefore, what is common to participants in particular activities, e.g. for surfers, might not only be the ocean as a material environment or the variety of waves as affordances but also the being of individuals embedded in human sociocultural practices, i.e. sharing stable ways of doing things and interpreting actions and living with others [67]. In the case of humans (or surfers in particular), these regular patterns, being manifested in sociocultural practices, can be exemplified by the emergence of multitude of participation styles and philosophies in AAS, i.e. varying styles of surfing and related lifestyles or different ‘forms of life’ in interacting with specific environmental conditions of the ocean, constrained by winds, surf, currents and proximity of other surfers.

Thus, a niche is a set of affordances which offers an individual a specific way of life [62]. An important notion when considering how distinguishable niches or disciplines are being evolved is that stable ways of doing things might surpass the boundaries of tool-related constraints. For example, the ‘ecological niche of free riders’ (big-mountain skiers and snowboarders) might have more in common in terms of effectivities that shape participation and experiences than effectivities common for big-mountain and freestyle snowboarders (two groups of snowboarders representing different forms of life). Therefore, ignoring sociocultural constraints when defining sports fails to capture the depth and nuances through which participation of individuals or groups in sports can be described.

When varying sociocultural practices evolve in similar environments, participants can acquire abilities that flourish in different practices from one’s own [67]. New possibilities for action can emerge for individuals in a material environment, simultaneously transcending the ‘sport’ itself. This idea exemplifies one potential constraint: interacting individual and social factors which can lead to the emergence of novel forms of movement such as ski-mountaineering or tow-in surfing, examples of humans making new combinations by exploring different fields in the rich landscape of affordances that the environment already offers. The important point here is that these innovations are an inherent aspect of AAS which has been completely ignored in previous research. They are predicated on the perception and exploitation of affordances which depend on effectivities, individually perceived by innovating athletes. Further, the development of new inventions, such as equipment (e.g. wingsuits) or built environments (e.g. cable wakeboarding parks, parkour facilities), extends the opportunities for exploiting rich possibilities for action available in a form of life, unveiling creative behaviours and forming previously unseen niches.

#### Skill Acquisition and Creativity in AAS

Although some attempts have been made, skilled performance in AAS should not be evaluated comprehensively by traditional quantitative measurements (e.g. measuring time, distance or score) or in comparison to other participants as in many traditional sports. In AAS particularly, aesthetic aspects such as personality of ‘style’ (i.e. how it looks and meets task goals) and creativity (i.e. innovation, novelty, originality) play a major role in how performances are judged [61, 68, 69] and further promoted [70] in an AAS form of life [61, 68, 69]. Here, we argue that this visual judgment, dependent on the perspective of other participants, represents a unique social constraint, interacting with each individual’s physical and psychosocial experiences (i.e. how it feels) [17, 47, 70]. In AAS, the functionality of skill (i.e. how effectively task goals are achieved) partially depends on subtle interactions of task and personal constraints such as originality, collective agreement and interpretation. Thus, an important task constraint to address is the value placed on creativity and innovation of actions expressed during performance or in terms of the contexts within which performance takes place. These notions further emphasize the need for a multidisciplinary approach and careful evaluation of research methods to understand these emerging characteristics shaping participation in AAS.

Central to any AAS is the innovative and creative behaviours of individuals or groups that compose a form of life. Sociocultural tendencies within forms of life, or ‘the field of promoted actions’ [70], can significantly constrain the behaviours of individuals, or reinforce established ones. However, particular social agreement among participants might, for example, invite them to act functionally, by challenging rules and norms. Therefore, development of expertise requires that a participant is not only well aware of underpinning sociocultural constraints or functionality of practice (i.e. usefulness, effectiveness, appropriateness or adequacy) but also knows how to diverge from them with novelty (i.e. originality). Functional skill (engaging with affordances to achieve goal-directed outcomes) might consequently consist of understanding and balancing actions relative to sociocultural influences that can be in conflict with personal style and tendencies. Performance is not predicated only on participation purely as subordinate to social norms but also on tendencies to imagine, innovate and explore action possibilities outside of (affordances available to) a particular niche. The culture of innovation in many AAS forms of life would suggest that behaviours supporting the capacity to explore new environments (e.g. urban skiing) and creative movement solutions (e.g. ‘tricks’ in skateboarding) are particularly functional to participants. Thus, in the case of AAS, creativity can be defined as ‘the process of perceiving, exploiting, and ‘generating’ novel affordances during socially and materially situated activities’ [71]. One practical implication of this idea is that (with solid understanding of sociocultural affordances) constraints in learning contexts should be designed to support continuous functional exploration of performers [52].

## Conclusions

The ecological dynamics framework provides a rich multidisciplinary platform for comprehensive and nuanced understanding of participation and creative actions of AAS participants, which emerge in all kinds of imaginable and ever-changing environments. Instead of trying to determine the ‘traditional ethos’ of each sport or categorizing (sub)disciplines, we have argued that deeper understanding can be gained by considering participation as emergent and acknowledging that performer-environment relationships are shaped by dynamic interacting constraints that distinguish a niche, or a form of life in AAS.

Thus, researchers and practitioners should refine a nuanced understanding and consider a sociocultural focus on affordances to appraise these characteristics and constraints within and across AAS. This is important when choosing research methods or pedagogical approaches or when designing learning environments. A fundamental consideration in the study of AAS is that rather than being predetermined, affordances emerge from a dynamic form of life in a sociocultural context. Practical examples of the historical development patterns of many AAS suggest that it would be most useful for future research to clarify the exploration of affordances outside of particular ecological niches or ‘discipline-typical’ environments (physical and sociocultural). In terms of originality, more attention is needed to understand possibilities potentially provided by interactions between activity categories or ‘human’ and ‘non-human’ interactions, including becoming attuned to affordances via new technologies and emerging online performance demonstration sub-arenas in social media. To better understand the link between affordances and creativity in any AAS, more empirical research needs to be conducted from an ecological, multidisciplinary perspective and directed towards the use of introspective and qualitative methods.

## References

[1] Thorpe H, Wheaton B (2011). ‘Generation X Games’, action sports and the olympic movement: understanding the cultural politics of incorporation. Sociology.

[2] Pain MT, Pain MA (2005). Essay: risk taking in sport. Lancet.

[3] Monasterio E (2007). The risks of adventure sports/people. Alpinist.

[4] Self DR, De Vries HE, Findley CS, Reilly E (2006). Thrill seeking: the type T personality and extreme sports. Int J Sport Manag Mark.

[5] Olivier S (2006). Moral dilemmas of participation in dangerous leisure activities. Leis Stud.

[6] Fave AD, Bassi M, Massimini F (2003). Quality of experience and risk perception in high-altitude rock climbing. J Appl Sport Psychol.

[7] Simon J (2002). Taking risks: extreme sports and the embrace of risk in advanced liberal societies. Embracing risk: the changing culture of insurance and responsibility.

[8] Pizam A, Reichel A, Uriely N (2001). Sensation seeking and tourist behavior. J Hosp Leis Mark.

[9] Le Breton D (2000). Playing symbolically with death in extreme sports. Body Soc.

[10] Rinehart R. Emerging arriving sport: alternatives to formal sports. Handbook of sports studies. London: Sage; 2000:504–20.

[11] Breivik G (1996). Personality, sensation seeking and risk taking among Everest climbers. Int J Sport Psychol.

[12] Rossi B, Cereatti L (1993). The sensation seeking in mountain athletes as assessed by Zuckerman’s Sensation Seeking Scale. Int J Sport Psychol.

[13] Laurendeau J (2006). “He didn’t go in doing a skydive”: sustaining the illusion of control in an edgework activity. Sociol Perspect.

[14] Laurendeau J (2008). “Gendered risk regimes”: a theoretical consideration of edgework and gender. Sociol Sport J.

[15] Hunt JC (1996). Diving the wreck: risk and injury in sport scuba diving. Psychoanal Q.

[16] Clough P, Mackenzie SH, Mallabon L, Brymer E (2016). Adventurous physical activity environments: a mainstream intervention for mental health. Sports Med.

[17] Brymer E, Downey G, Gray T (2009). Extreme sports as a precursor to environmental sustainability. J Sport Tour.

[18] Brymer E, Oades LG (2008). Extreme sports: a positive transformation in courage and humility. J Humanist Psychol.

[19] Willig C (2008). A phenomenological investigation of the experience of taking part in ‘Extreme Sports’. J Health Psychol.

[20] Allman TL, Mittelstaedt RD, Martin B, Goldenberg M (2009). Exploring the motivations of BASE jumpers: extreme sport enthusiasts. J Sport Tour.

[21] Brymer E, Schweitzer R (2013). The search for freedom in extreme sports: a phenomenological exploration. Psychol Sport Exerc.

[22] Brymer E, Gray T (2009). Dancing with nature: rhythm and harmony in extreme sport participation. J Adv Educ Outdoor Learn.

[23] Brymer E (2009). The role of Extreme Sports in lifestyle enhancement and wellness. Proceedings of the 26th ACHPER International Conference: Creating Active Futures.

[24] Lima R, Rosa G, Braga De Mello D, Albergaria M, Fernandes Filho J (2011). Cardiovascular parameters and body composition of professional female surfers. Int Sport Med J.

[25] Kerr JH, Mackenzie SH (2012). Multiple motives for participating in adventure sports. Psychol Sport Exerc.

[26] Brymer E, Schweitzer R (2012). Extreme sports are good for your health: a phenomenological understanding of fear and anxiety in extreme sport. J Health Psychol.

[27] Mackenzie SH, Hodge K, Boyes M (2011). Expanding the flow model in adventure activities: a reversal theory perspective. J Leis Res.

[28] Gerber M, Kalak N, Lemola S, Clough PJ, Pühse U, Elliot C (2012). Adolescents’ exercise and physical activity are associated with mental toughness. Ment Health and Phys Act.

[29] Brymer E (2009). Extreme sports as a facilitator of ecocentricity and positive life changes. World Leis J.

[30] Brymer E, Schweitzer RD. Phenomenology and extreme sports in natural landscapes. In: landscapes of leisure. Springer; 2015. p. 135–46.

[31] Malone K. The bubble-wrap generation: children growing up in walled gardens. Environ Educ Res. 2007;13(4):513–27. doi:10.1080/13504620701581612.

[32] Wheaton B (2015). Assessing the sociology of sport: on action sport and the politics of identity. Int Rev Sociol Sport.

[33] Thorpe H, Wheaton B. Dissecting action sports studies. A companion to sport. 2013:341–58.

[34] Davids K, Brymer E, Seifert L, Orth D. 18 A constraints-based approach to the acquisition of expertise in outdoor adventure sports. Complex Syst Sport. London: Routledge; 2013;7:306.

[35] Breivik G (2010). Trends in adventure sports in a post-modern society. Sport Soc.

[36] Brymer E (2010). Risk taking in Extreme Sports: A phenomenological perspective. Ann Leis Res.

[37] Booth D, Thorpe H (2007). International encyclopedia of extreme sport.

[38] Gilchrist P, Wheaton B (2011). Lifestyle sport, public policy and youth engagement: examining the emergence of parkour. Int J Sport Policy Politics.

[39] Wheaton B (2004). Understanding lifestyle sport: consumption, identity and difference.

[40] Wheaton B (2010). Introducing the consumption and representation of lifestyle sports.

[41] Krein K (2008). Sport, nature and worldmaking. Sports Ethics Philos.

[42] Thorpe H, Rinehart R (2010). Alternative sport and affect: non-representational theory examined. Sport Soc.

[43] Barlow M, Woodman T, Hardy L (2013). Great expectations: different high-risk activities satisfy different motives. J Pers Soc Psychol.

[44] Woodman T, Barlow M, Bandura C, Hill M, Kupciw D, Macgregor A (2013). Not all risks are equal: the risk taking inventory for high-risk sports. J Sport Exerc Psychol.

[45] Langseth T (2011). Risk sports–social constraints and cultural imperatives. Sport Soc.

[46] Thomson CJ, Power RJ, Carlson SR, Rupert JL, Michel G (2015). A comparison of genetic variants between proficient low- and high-risk sport participants. J Sports Sci.

[47] Varley PJ (2011). Sea kayakers at the margins: the liminoid character of contemporary adventures. Leis Stud.

[48] Creyer E, Ross W, Evers D (2003). Risky recreation: an exploration of factors influencing the likelihood of participation and the effects of experience. Leis Stud.

[49] Online Etymology Dictionary [Internet].; 2016 []. Available from: http://www.etymonline.com/index.php?term=sport. Accessed 19 Nov 2016.

[50] KOTUS Kotimaisten kielten keskus. Kielitoimiston sanakirja [Internet].; 2017 []. Available from: http://www.kielitoimistonsanakirja.fi/netmot.exe?motportal=80. Accessed 19 Nov 2016.

[51] Atkinson M (2010). Entering scapeland: yoga, fell and post-sport physical cultures. Sport Soc.

[52] Seifert L, Orth D, Button C, Brymer E, Davids K. An ecological dynamics framework for the acquisition of perceptual-motor skills in climbing. In: Extreme Sports Medicine. Springer; 2017. p. 365–82.

[53] Davids KW, Button C, Bennett SJ. Dynamics of skill acquisition: a constraints-led approach. Champaigne, IL: Human Kinetics. Human Kinetics; 2008.

[54] Newell KM. Constraints on the development of coordination. Motor development in children: aspects of coordination and control. Dordrecht: Martinus Nijhoff. 1986;34:341–60.

[55] Orth D, Davids K, Araújo D, Renshaw I, Passos P. Effects of a defender on run-up velocity and ball speed when crossing a football. Eur J Sport Sci. 2014;14(sup1):S316–23. doi: http://dx.doi.org/10.1080/17461391.2012.696712. 10.1080/17461391.2012.696712 24444224

[56] Greenwood D, Davids K, Renshaw I. The role of a vertical reference point in changing gait regulation in cricket run-ups. Eur J Sport Sci. 2016:1–7. 10.1080/17461391.2016.1151943 26902778

[57] Cordovil R, Araujo D, Davids K, Gouveia L, Barreiros J, Fernandes O (2009). The influence of instructions and body-scaling as constraints on decision-making processes in team sports. Eur J Sport Sci.

[58] Lawrence GP, Cassell VE, Beattie S, Woodman T, Khan MA, Hardy L (2014). Practice with anxiety improves performance, but only when anxious: evidence for the specificity of practice hypothesis. Psychol Res.

[59] Sanchez X, Boschker M, Llewellyn D (2010). Pre‐performance psychological states and performance in an elite climbing competition. Scand J Med Sci Sports.

[60] Brymer E, Schweitzer R (2013). Extreme sports are good for your health: a phenomenological understanding of fear and anxiety in extreme sport. J Health Psychol.

[61] Ojala A. Vaihtoehtoisuutta ja valtavirtaisuutta: tutkimus suomalaisten lumilautailijoiden uria raamittavista asenteista, olosuhteista ja resursseista. 2015.

[62] Gibson JJ, Bray R. 1986. The ecological approach to visual perception. Hillsdale: Erlbaum; 1979.

[63] Davids K, Araújo D, Brymer E. Designing Affordances for health-enhancing physical activity and exercise in sedentary individuals. Sports Medicine. 2016:1–6. doi:10.1007/s40279-016-0511-3. 10.1007/s40279-016-0511-3 26914265

[64] Shaw R, Turvey MT, Mace W. Ecological psychology: the consequence of a commitment to realism. Cognition and the symbolic processes. Hillsdale: Erlbaum. 1982;2:159–226.

[65] Wittgenstein L. 2001. Philosophical investigations. Oxford: Blackwell; 1953;3.

[66] Wittgenstein L. Lectures on Aesthetic. Oxford: Blackwell; 1978.

[67] Rietveld E, Kiverstein J (2014). A rich landscape of affordances. Ecol Psychol.

[68] Ojala A (2014). Institutionalisation in professional freestyle snowboarding-Finnish professional riders’ perceptions. Eur J Sport Soc.

[69] Thorpe H. Action sports, social media, and new technologies towards a research agenda. Communication & Sport. 2016:2167479516638125.

[70] Reed E, Brill B. The primacy of action in development, in Latash & Turvey. Dexterity and its Development). Mahwah: LEA. Taylor & Francis; 1996.

[71] Glăveanu VP. What can be done with an egg? Creativity, material objects, and the theory of affordances. J Creat Behav. 2012;46(3):192–208. Wiley Online Library. doi:10.1002/jocb.13.

[72] Boden MA. Dimensions of creativity. MIT Press; 1996.

[73] Hristovski R, Davids K, Araujo D, Passos P (2011). Constraints-induced emergence of functional novelty in complex neurobiological systems: a basis for creativity in sport. Nonlinear Dynamics Psychol Life Sci.

[74] Thorpe H (2009). Understanding ‘alternative’sport experiences: a contextual approach for sport psychology. Int J Sport Exerc Psychol.

[75] Brymer GE. Extreme dude! A phenomenological perspective on the extreme sport experience. 2005

[76] Brymer E. The extreme sports experience: a research report. IFPRA world. 2009:6–7. Reading, UK: IFPRA.

[77] Yeh H, Stone JA, Churchill SM, Wheat JS, Brymer E, Davids K. Physical, psychological and emotional benefits of green physical activity: an ecological dynamics perspective. Sports Med. Basel: Springer International Publishing; 2015:1–7. 10.1007/s40279-015-0374-z 26330207

[78] Kerr JH, Mackenzie SH (2014). Confidence frames and the mastery of new challenges in the motivation of an expert skydiver. Sport Psychologist.

